# Reorganisation of the Salivary Mucin Network by Dietary Components: Insights from Green Tea Polyphenols

**DOI:** 10.1371/journal.pone.0108372

**Published:** 2014-09-29

**Authors:** H. Davies-Strickleton, Paul D. A. Pudney, Pantelis Georgiades, Thomas A. Waigh, Nigel W. Hodson, Caroline E. Ridley, Ewan W. Blanch, David J. Thornton

**Affiliations:** 1 Wellcome Trust Centre for Cell-Matrix Research, Faculty of Life Sciences, University of Manchester, Manchester, United Kingdom; 2 Manchester Institute of Biotechnology, Faculty of Life Sciences, The University of Manchester, Manchester, United Kingdom; 3 Unilever Discover, Shambrook, Bedfordshire, United Kingdom; 4 Biological Physics, Department of Physics and Astronomy, University of Manchester, Manchester, United Kingdom; 5 BioAFM Facility Centre for Tissue Injury and Repair, Faculty of Medical and Human Sciences, University of Manchester, Manchester, United Kingdom; University of South Florida College of Medicine, United States of America

## Abstract

The salivary mucins that include MUC5B (gel-forming) and MUC7 (non-gel-forming) are major contributors to the protective mucus barrier in the oral cavity, and it is possible that dietary components may influence barrier properties. We show how one dietary compound, the green tea polyphenol epigallocatechin gallate (EGCG), can substantially alter the properties of both the polymeric MUC5B network and monomeric MUC7. Using rate-zonal centrifugation, MUC5B in human whole saliva and MUC5B purified from saliva sedimented faster in the presence of EGCG. The faster sedimentation by EGCG was shown to be greater with increasing MUC5B concentration. Particle tracking microrheology was employed to determine the viscosity of purified MUC5B solutions and showed that for MUC5B solutions of 200–1600 µg/mL, EGCG caused a significant increase in mucin viscosity, which was greater at higher MUC5B concentrations. Visualisation of the changes to the MUC5B network by EGCG was performed using atomic force microscopy, which demonstrated increased aggregation of MUC5B in a heterogeneous manner by EGCG. Using trypsin-resistant, high-molecular weight oligosaccharide-rich regions of MUC5B and recombinant N-terminal and C-terminal MUC5B proteins, we showed that EGCG causes aggregation at the protein domains of MUC5B, but not at the oligosaccharide-rich regions of the mucin. We also demonstrated that EGCG caused the majority of MUC7 in human whole saliva to aggregate. Furthermore, purified MUC7 also underwent a large increase in sedimentation rate in the presence of EGCG. In contrast, the green tea polyphenol epicatechin caused no change in the sedimentation rate of either MUC5B or MUC7 in human whole saliva. These findings have demonstrated how the properties of the mucin barrier can be influenced by dietary components. In the case of EGCG, these interactions may alter the function of MUC5B as a lubricant, contributing to the astringency (dry puckering sensation) of green tea.

## Introduction

Saliva is the body’s first line of defence to ingested insults, such as pathogens and chemicals, and is paramount to the protection of hard and soft tissues in the oral cavity and alimentary canal [1]–[4]. This complex barrier is composed of many components, including the high-molecular weight, heavily O-glycosylated gel-forming mucin MUC5B, which forms the viscoelastic network that is important for hydration, lubrication, pathogen exclusion and resistance to proteolytic digestion. Other salivary components, including cystatins, histatins, immunoglobulins, proline-rich proteins and the small non-gel-forming mucin MUC7, have functions in innate immunity, which may be facilitated by the formation of heterotypic complexes [5]–[10].

Altered saliva composition or salivary gland hypofunction, is often caused by medications used to treat head and neck cancers. The aberrant saliva can result in symptoms of xerostomia (dry mouth) including dryness, pain and discomfort, and this may also interfere with oral defence [11]–[13]. The relief of some of these symptoms by treatment with mucin-based saliva substitutes highlights the importance of the mucin-rich gel network in saliva [14], [15]. In addition to disrupted saliva integrity during disease, the composition and properties of saliva also varies in healthy individuals since the secretion and protein content of saliva have been shown to vary with time of the day and can be influenced by lifestyle [16], [17]. It is possible, therefore, that diet may alter the structure and function of salivary components. We explore this possibility with one type of dietary compound, green tea polyphenols.

There has been considerable interest in plant polyphenols since they are reported to have protective roles against cancer and heart disease, owing to their anti-oxidant and anti-carcinogenic activities [18], [19]. However, polyphenols have poor bioavailability, and contribute to the astringency (dry puckering sensation in the oral cavity) of polyphenol containing foods and beverages, such as green tea. For the major polyphenol in green tea, epigallocatechin gallate (EGCG), these effects are thought to be due partly to the interactions of polyphenols with salivary components, which may influence the properties of both interaction partners [20], [21]. In contrast, the astringent properties of another polyphenol in green tea, epicatechin (EC), may not be due to polyphenol-altered properties of saliva [20], [22]. For EGCG, the various salivary proteins that have been extensively reported to interact with this polyphenol include proline-rich proteins, cystatins, histatins and amylase [23]–[29]. Surprisingly, much less is known about the interactions of the main structural component of saliva, the polymeric gel-forming mucin MUC5B [30], and non-gel-forming mucin, MUC7 [31] with green tea polyphenols.

There are contrasting reports on the effect of EGCG on salivary mucins. Proteomic studies of human whole saliva mixed with different polyphenols suggest that mucins are not among those proteins precipitated by polyphenols [28], [32], [33], while, in contrast, studies on poorly characterised commercially available mucins report their aggregation by polyphenols [34]–[37]. A more recent study on a well characterised polymeric porcine gastric mucin, Muc5ac, has shown an increase in mucin viscosity in the presence of EGCG [38], but it has not been demonstrated if a similar response occurs for MUC5B in saliva, the first mucosal secretion encountered by ingested foods. Moreover, the mechanism of mucin aggregation by EGCG is undetermined, and is part of the focus of this investigation.

Investigation of well characterised mucins purified from human whole saliva would enable elucidation of the contribution of the salivary mucins to the limited bioavailability and astringency of green tea polyphenols, and would also provide insights into our understanding of how dietary components influence saliva integrity at the level of the mucin network. This could have consequences for the functions of MUC5B and MUC7 in saliva and for mucosal barriers throughout the gastrointestinal tract, including altered pathogen binding and formation of immune complexes, and altered network properties that may affect the absorption of nutrients in the small intestine.

In this study, we examined the interaction of EGCG (over a concentration range typically found in a cup of green tea [18], [39]) with MUC5B and MUC7 in human whole saliva, and MUC5B and MUC7 purified from human whole saliva by isopycnic density gradient centrifugation. We investigated the sedimentation behaviour of MUC5B and MUC7 using rate-zonal centrifugation, to determine mucin structural changes in the presence of EGCG. Moreover, the consequences of EGCG on the properties of the MUC5B network were examined by atomic force microscopy (AFM) and particle tracking microrheology (PTM). The location of EGCG binding sites on MUC5B was explored by rate-zonal centrifugation of highly glycosylated regions of MUC5B (T-domains, isolated by trypsin digestion) and expressed recombinant proteins of the full N-terminal and C-terminal protein domains of MUC5B with EGCG [40]. We found that at concentrations of EGCG typical of green tea, there are substantial changes to the network properties of MUC5B and MUC7, which may impact both saliva organisation and green tea polyphenol bioavailability and astringency. In marked contrast, EC caused no change in the sedimentation rate of MUC5B or MUC7.

## Materials and Methods

### Materials

Sequencing-grade modified trypsin was purchased from Promega. Stock 8 M guanidinium chloride (GdmCl) was prepared by charcoal filtration of GdmCl (Sigma-Aldrich). Stock 6 M urea was prepared by charcoal filtration of urea (VWR) followed by deionisation with Amberlite mixed bed resin (Sigma-Aldrich). Stock solutions of sucrose were treated with charcoal before use. Agarose, caesium chloride (CsCl) and dithiothreitol (DTT) were purchased from Melford. The EGCG used was commercially available (Teavigo), DSM Nutritional Products Inc., purified from green tea. The sample was found to be 94% EGCG by HPLC. EC was isolated from green tea and found to be 98.4% pure by HPLC [20].

### Ethics statement

Saliva was donated by healthy participants with no overt sign of oral pathologies and who provided their written consent. Donors refrained from eating or drinking for at least 1 hour prior to collection and saliva was collected in a sterile container that was kept on ice during collection. For experiments using human whole saliva, donations were used immediately after collection. For mucin purification, saliva was immediately solubilised, as described below. Ethical approval for this research was acquired from the University of Manchester.

### Purification and characterisation of salivary mucins

Human whole saliva was pooled and the mucins were purified by isopycnic density gradient centrifugation [41]. MUC5B was purified under non-denaturing conditions, whereas MUC7 was purified under denaturing conditions. Saliva was solubilised overnight in an equal volume of 0.2 M NaCl with protease inhibitors (non-denaturing) or 8 M GdmCl (denaturing) at 4°C. Mucins were subjected twice to density gradient centrifugation in CsCl/4 M GdmCl (starting density 1.4 g/mL) followed by density gradient centrifugation in CsCl/0.2 M GdmCl (starting density 1.5 g/mL) in a Beckman L-90 ultracentrifuge (Beckman Ti45 rotor, 72 hours, 40000 rpm, 15°C). For non-denaturing purification, 0.1 M NaCl was used in place of GdmCl.

Following denaturing isopycnic density gradient centrifugation, MUC7 was purified further by size exclusion chromatography on a Sepharose CL-2B column (80 cm x 23 mm) eluted with 4 M GdmCl.

Mucin purity and identify was analysed using mass spectrometry [42]. Briefly, sample preparations were digested with trypsin, and tryptic peptides were recovered free from salts using a C-18 ZipTip (Millipore). Samples were analysed by LC-MS/MS using a NanoAcquity LC coupled to a LTQ Velos mass spectrometer. In the MS/MS analysis of purified polymeric MUC5B, MUC5B was identified as the most abundant protein by comparison of the data with the Uniprot KB protein database using the MASCOT (version 2.2) search engine. While a small number of other proteins were identified, the number of unique tryptic peptides identified in these proteins was very low compared to MUC5B, demonstrating that the vast majority of salivary proteins had been removed. MS/MS analysis of purified MUC7 confirmed the presence of MUC7 as the major component, and also identified MUC5B and a small number of other salivary proteins, however, light scattering analysis showed the most abundant species had a molecular weight corresponding to MUC7 (described below). There was no evidence for the presence of proline-rich proteins in either of the MUC5B or MUC7 samples.

Mucin molecular weight distribution was analysed using multi-angle laser light scattering with size exclusion chromatography (SEC/MALLS) according to well established protocols [43]. Polymeric MUC5B was separated on Shodex columns connected in series (OHpk SBG (Guard column), OHpak SB-806M HQ (exclusion limit 20 MDa, 8 mm x 300 mm), OHpak SB-807 HQ (exclusion limit 500 MDa, 8 mm x 300 mm)), and was found to have a molecular weight range from 5–40 MDa, with an average molecular weight of 26 MDa. MUC7 was analysed by SEC/MALLS on a Superose 6 column (30 cm x 10 mm), in 200 mM NaCl, 1 mM EDTA and was found to have an average molecular weight of 240 kDa.

### Generation of MUC5B T-domains

MUC5B was reduced, alkylated and subjected to trypsin digestion to yield highly glycosylated T-domains, based on protocols established previously [44]. Purified mucins were buffer exchanged into 0.1 M TrisHCl, pH 8 using prewashed Sartorious Vivaspin 5 or 10 kDa MWCO columns. Samples were reduced at a final concentration of 20 mM DTT for 37°C for 3 hours, alkylated with 50 mM iodoacetamide (IAA) for 45 minutes in the dark at room temperature and the buffer was exchanged into 0.1 M TrisHCl pH8 for trypsin digestion. Trypsin was added at a ratio of 1∶50 (trypsin: protein) at 37°C overnight. Centrifugation in Sartorious Vivaspin 5 or 10 kDa MWCO columns retained trypsin resistant T-domains, which were buffer exchanged into PBS, pH 7.4 for rate-zonal centrifugation. Mucin T-domains were analysed by SEC/MALLS by separation on a Superose 6 column (30 cm x 10 mm), in 200 mM NaCl, 1 mM EDTA; two species were identified with average molecular weights of 240 and 550 kDa.

### Expression and purification of recombinant human MUC5B N-terminal and C-terminal domains

An N-terminal construct, consisting of D1-D2-D′-D3 domains of MUC5B (NT5B, residues 26-1304), was created and recombinant proteins expressed with an N-terminal His6 tag using a pCEP-HIS vector in stably transfected 293-EBNA cells [40]. Conditioned media was collected and recombinant protein purified by nickel-affinity chromatography using a 1 mL HisTrap FF column (GE Healthcare) followed by size exclusion chromatography on Superose 6 (10/300 column eluted in 25 mM Tris/10 mM NaCl pH 7.4) and anion exchange chromatography on a 1 mL Resource Q column (eluted with a gradient of 0–0.5 M NaCl in 25 mM Tris pH 7.4).

A C-terminal construct, consisting of D4-B-C-CK domains of MUC5B (CT5B, residues 4958–5765) was created and recombinant proteins were expressed and purified under the same conditions.

### Rate-zonal centrifugation

Rate-zonal centrifugation was performed as described previously [45]. Linear sucrose gradients of 10–50% (w/v) (human whole saliva), 10–35% (w/v) (polymeric MUC5B) or 5–20% (w/v) (MUC5B T-domains, NT5B, CT5B and MUC7) were prepared in PBS pH 7.4 in 14 mL Beckman Coulter polyallomer centrifuge tubes using a gradient maker connected to a Gilson Minipuls 2 multichannel peristaltic pump (Anachem). The sample (500 µL: 4 parts saliva/mucin/mucin fragment and 1 part polyphenol (various concentrations to give final EGCG concentrations between 0.1–4 mM or a final EC concentration of 1 mM)) was layered on to the gradient and centrifuged in a Beckman L-90 ultracentrifuge (Beckman swing out SW40 rotor, 40000 rpm, 15°C) for 1 hour (human whole saliva), 1 hour 15 minutes (polymeric MUC5B) or 2 hour 30 minutes (MUC5B T-domains, NT5B, CT5B and MUC7). Sucrose solutions used for MUC5B T-domains, NT5B, CT5B and MUC7 gradients had EGCG added so that a final concentration of 4 mM EGCG was present throughout the gradient, for determination of whether interactions occurred with EGCG in excess. Gradients were fractionated from the top and pelleted material recovered by solubilisation in urea. The distribution of mucins/mucin fragments was determined by agarose gel electrophoresis with western blotting (intact mucins), SDS-PAGE (NT5B and CT5B) or PAS slot blot analysis (MUC5B T-domains).

### PAS slot blot

Prior to PAS slot blotting, rate-zonal centrifugation fractions were separated from EGCG on a 5 mL HiTrap desalting column (GE healthcare). Samples were loaded onto nitrocellulose membranes using Minifold II 72 well slot blot apparatus with a water suction vacuum.

Nitrocellulose membranes were washed briefly with ddH_2_O, incubated with 1% (v/v) periodic acid/3% (v/v) acetic acid for 30 minutes, washed with ddH_2_O (for 3×5 minutes), washed with 0.1% (w/v) sodium metabisulphite/10 mM HCl (for 3×5 minutes) and developed in Schiff’s reagent for 10–30 minutes [46]. Development was stopped with 0.1% (w/v) sodium metabisulphite/10 mM HCl. Blots were then rinsed with ddH_2_O, dried and scanned images were captured using a Biorad ChemiDoc MP Imaging System with Image Lab software (version 4.1).

### Agarose gel electrophoresis and western blotting

Horizontal gel electrophoresis was performed in Bio-Rad Subcell gel tanks with 0.7% (w/v) agarose gels in TAE buffer (20 mM Tris-base/1 mM EDTA/0.1% (w/v) SDS, pH 8) at 65 V for 3–6 hours. Fractions were loaded in equal volumes to allow comparison of band staining intensities. Gels of unreduced samples were reduced in 10 mM DTT for 20 minutes prior to transfer to nitrocellulose. Proteins were transferred from gels to nitrocellulose membrane using Pharmacia LKB VacuGene XL vacuum blotting apparatus connected to a VacuGene pump (GE Healthcare) in 0.6 M NaCl, 60 mM sodium citrate, pH 8 for 2 hours at a suction pressure of 40–60 mbar [47].

Membranes were blocked with 4% (w/v) milk in PBS and incubated with primary antibody in TBST (anti-MUC5B polyclonal antibody MAN-5BIII raised against the sequence CSWYNGHRPEPGLG [48]; anti-MUC7 monoclonal antibody EUMUC7A raised against the peptide EGRERDHELRHRRHHHQ [49]) overnight. After washing, blots were probed with a secondary antibody (IRDye 800 goat anti-rabbit or IRDye 680RD goat anti-mouse) and imaged using the LI-COR Odyssey CLx infrared imaging system. Band intensities were measured using the Odyssey software (version 2.1) with the average background method and border width of 3 pixels all around the selected features.

### SDS-PAGE

Electrophoresis was performed on 4–12% (w/v) RunBlue Bis-Tris precast gels (Expedeon), with RunBlue rapid SDS run buffer (Expedeon) at 180 V for 1 hour. Gels were silver stained (NT5B) or Coomassie stained (CT5B), and imaged using a Biorad ChemiDoc MP Imaging System. Band intensities were measured using Image Lab software (version 4.1).

### Atomic force microscopy (AFM)

Freshly cleaved mica was silanized with 60 µL 0.03% (v/v) aminopropyl-triethoxysilane (APTES) in a humidity chamber (pipette tip box) for 30 minutes, rinsed with ddH_2_O (3×300 µL) and air dried for 2 hours. MUC5B (15 µg/mL) purified by isopycnic density gradient centrifugation, was dialysed extensively in PBS pH 7.4 and incubated in solution at a ratio of 5∶1 with PBS (untreated MUC5B). For EGCG-treated MUC5B, purified MUC5B (15 µg/mL) was incubated in solution at a ratio of 5∶1 with EGCG, to give final concentrations of 75 µM and 375 µM EGCG. Samples (50 µL) were added to APTES-coated mica for 30 seconds. Non-absorbed sample was removed by washing with ddH_2_O (6×300 µL) and samples were air dried overnight.

Samples were imaged by intermittent contact mode in air using a Bruker Multimode AFM with a Nanoscope V controller and a “J” scanner. Imaging was performed using Olympus high aspect ratio etched silicon probes (OTESPA) with nominal spring constant of 42N/m (Bruker AXS S.A.S, France). Cantliever oscillation varied between 300 and 350 kHz, whilst the drive amplitude was determined by the Nanoscope (version 8.15) software. The set-point was adjusted to just below the point at which tip-sample interaction was lost. Height, phase and amplitude images with scan sizes of 5 µm^2^ were captured at a scan rate of 1 Hz and a relative humidity of <40%. WSxM software was used for image processing [50]. Particle analysis was performed in Nanoscope Analysis software (version 1.40) after images were flattened with a first-order polynomial fit. Measurements were analysed by parametric paired t-tests in GraphPad prism (version 6).

### Particle tracking microrheology (PTM)

MUC5B purified by isopycnic density gradient centrifugation was buffer exchanged into 0.1 M NaCl. Samples were mixed 5∶1 with EGCG to provide a final concentration of 1 mM EGCG. Uncoated beads were added to samples and PTM was carried out according to [38], with the following exceptions: videos were recorded at 500 frames per second for 1000 frames at a resolution of 1024×512 pixels; fifteen videos were recorded per sample, with four different preparations made for each sample in order to perform statistical analysis. Two-dimensional bead trajectories were acquired from videos, and trajectories converted to mean square displacements (MSDs) using our PolyParticle Tracker Matlab software [38], [51]. The MSD, 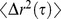, is dependent on lag time, 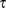, with proportionality constant, 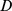 (the diffusion coefficient):
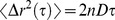
(1)where 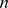 is the number of dimensions (i.e. equal to 2 in conventional optical microscopy). For purely viscous and viscoelastic fluids 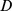 can be calculated from the fluctuation dissipation theorem:
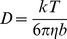
(2)where 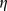 is the viscosity, 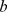 is the bead radius and 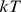 is the thermal energy. The MSD curve can be converted to the time dependent compliance, 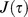, a standard measure of viscoelasticity [52] using:


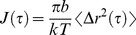
(3)Application of the Maxwell model of linear viscoelasticity relates 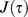 to viscosity and elastic modulus 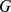 by the equation:
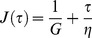
(4)

Viscosity measurements were analysed by parametric paired t-tests in GraphPad prism (version 6).

## Results

### The polymeric MUC5B network is altered by EGCG

**Figure 1:**
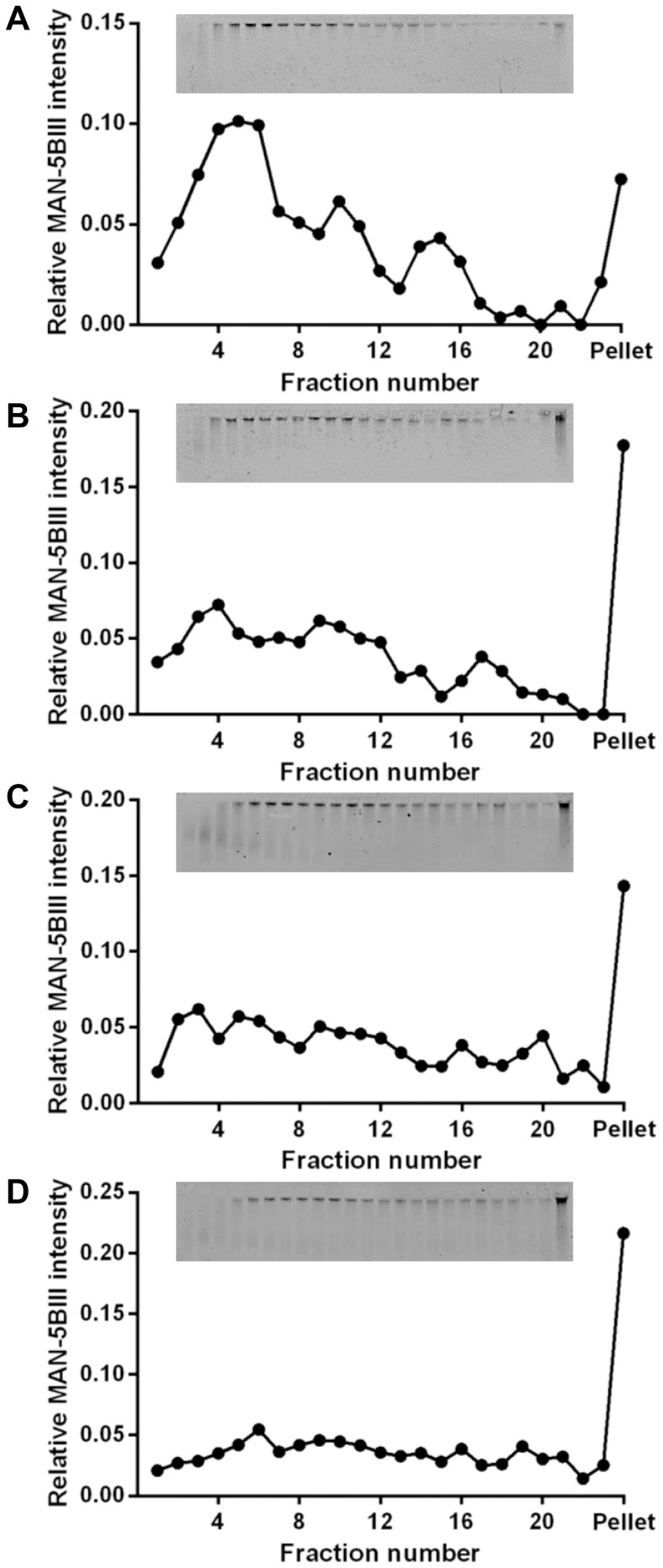
MUC5B in human whole saliva sediments faster in the presence of EGCG. Human whole saliva was incubated with (A) PBS, (B) 0.5 mM EGCG, (C) 1 mM EGCG or (D) 4 mM EGCG and subjected to rate-zonal centrifugation in 10–50% (w/v) sucrose gradients for 1 hour. MUC5B was detected by agarose gel electrophoresis and western blotting with the MAN-5BIII antiserum (insets). Bands were quantified using the Odyssey Imaging system.

Human whole saliva was subjected to rate-zonal centrifugation on 10–50% (w/v) sucrose gradients. MUC5B was detected in fractions by western blotting after agarose gel electrophoresis, and the band intensities were quantified (Figure 1). In the absence of EGCG, MUC5B was largely contained within the fractions in the top half of the gradient and only a small proportion pelleted (Figure 1 A).

Saliva was treated with different concentrations of EGCG. The amount of EGCG in a cup of green tea is variable, ranging from 0.3–1.8 mM [18], [39], [53]. Therefore, 1 mM EGCG was used here, as well as concentrations above and below this level to gain insight into the concentration dependence of its effect on mucin aggregation. The presence of 0.5, 1 and 4 mM EGCG in saliva (Figure 1 B, C and D, respectively), resulted in a broader MUC5B distribution in the sucrose gradient, and an increase in the sedimentation rate, as well as an increase in the amount pelleted compared to the control. Furthermore, a dose-dependency was observed, indicated by the increased proportion of MUC5B in the bottom half of the gradient, including the pellet, with increasing EGCG concentration. The faster sedimentation of MUC5B in the presence of EGCG suggests compaction or aggregation of MUC5B in saliva. To examine this further, native (non-GdmCl denatured) polymeric MUC5B was purified from human whole saliva.

**Figure 2:**
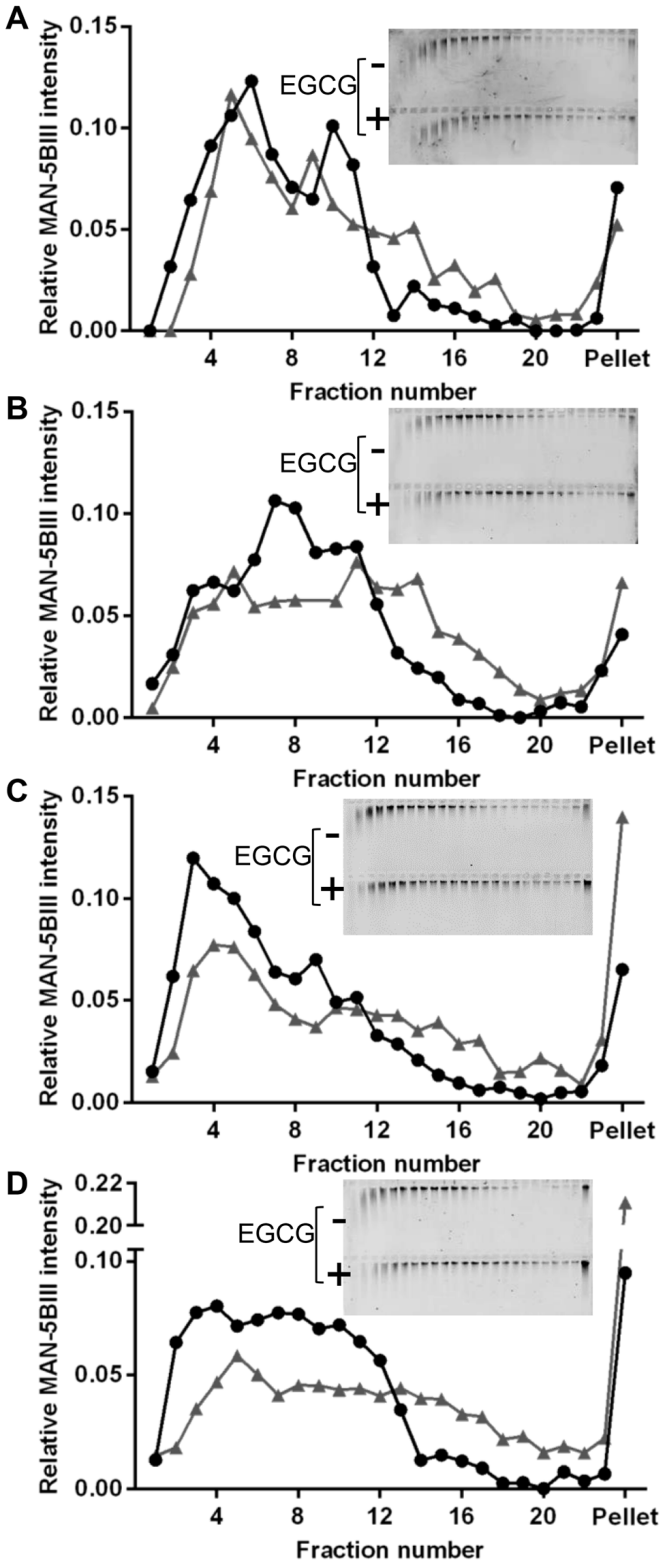
Increased sedimentation rate of purified MUC5B in the presence of 1 mM EGCG. Rate-zonal centrifugation of MUC5B (A: 200 µg/mL, B: 400 µg/mL, C: 800 µg/mL, D: 1.6 mg/mL) with 1 mM EGCG (grey triangles) or PBS (black circles), performed in 10–35% (w/v) sucrose gradients in PBS. MUC5B was detected by agarose gel electrophoresis and western blotting with the MAN-5BIII antiserum (insets; MUC5B (top panel) and MUC5B+EGCG (bottom panel)). Bands were quantified using the Odyssey Imaging system.

The effect of EGCG on the sedimentation behaviour of natively purified MUC5B was analysed by rate-zonal centrifugation on 10–35% (w/v) sucrose gradients. The concentration of MUC5B in saliva is highly variable between individuals as well as in different parts of the mouth; 200 µg/mL is reported to be the average MUC5B concentration in saliva, and this may be up to 5 times higher at the oral mucosal pellicle/salivary film that lines the soft tissues of the oral cavity [54]–[56]. At all concentrations of MUC5B studied (200–1600 µg/mL), the major proportion of untreated MUC5B sedimented in fractions 1–12 (Figure 2). Addition of 1 mM EGCG to all MUC5B solutions resulted in an increase in the proportion of MUC5B in the bottom half of the gradient. This effect was increased at higher MUC5B concentrations, suggesting the interaction is also dependent on mucin concentration (Figure 2 B–D). The faster sedimentation of MUC5B by EGCG suggests that the polyphenol may increase mucin molecular weight by inducing aggregation. To investigate the impact of EGCG on the properties of the MUC5B network, PTM was performed.

**Figure 3:**
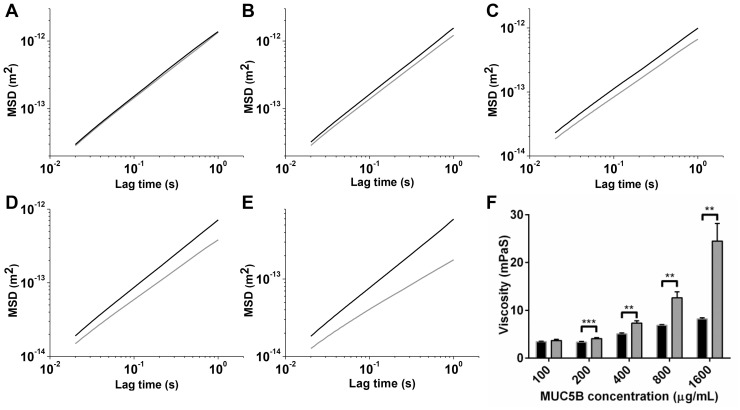
PTM of polystryrene beads in MUC5B and EGCG solutions. MSD of 505 nm polystyrene beads in mixtures of MUC5B (A: 100 µg/mL, B: 200 µg/mL, C: 400 µg/mL, D: 800 µg/mL, E: 1.6 mg/mL) with 1 mM EGCG (grey) or PBS (black). MSDs were derived from 15 videos per sample (four samples were measured for each mixture, n = 4). Representative ensemble MSDs are shown. F: MSDs were used to calculate viscosity for MUC5B (black) and MUC5B+EGCG mixtures (grey), (n = 4). The error bars show standard deviation. Parametric paired t-tests were performed to determine signficance; 200, 400, 800 and 1600 µg/mL were statistically significant compared to the control, with p values of 0.0007, 0.0017, 0.0039 and 0.0027 respectively. ** p value 0.001–0.01. *** p value 0.0001–0.001.

Using uncoated polystyrene beads (505 nm), PTM was carried out on MUC5B solutions in the absence and presence of 1 mM EGCG to determine the MSD of the beads (Figure 3). The graphs of MSDs as a function of lag time for untreated MUC5B showed shallower gradients with increasing mucin concentration, suggesting more restricted bead movement at higher MUC5B concentrations. Furthermore, as described in the Materials and Methods, MSD values were used to determine the viscosities, which showed that higher MUC5B concentration led to increased viscosity in untreated solutions (Figure 3 F). This concentration-dependent increase in MUC5B viscosity is due to greater mucin entanglement at higher concentrations. Upon the addition of 1 mM EGCG, an even greater increase in viscosity was observed.

At all MUC5B concentrations above 100 µg/mL the MSD curve of EGCG-treated MUC5B had a shallower slope than untreated MUC5B, demonstrating restricted movement of the beads. Furthermore, this restriction of bead movement in the presence of EGCG increased with MUC5B concentration (Figure 3 B–E). Using MSDs to calculate viscosity, it can be seen that EGCG treatment led to an increase in MUC5B viscosity at every MUC5B concentration tested. For MUC5B concentrations 200, 400, 800 and 1600 µg/mL the increased viscosity by EGCG was found to be statistically significant compared to the control. Furthermore, the increase in viscosity by EGCG was greater at higher MUC5B concentrations, with fold increases of 1.07 (100 µg/mL), 1.22 (200 µg/mL), 1.44 (400 µg/mL), 1.84 (800 µg/mL) and 2.98 (1600 µg/mL), clearly demonstrating the concentration-dependence of the effect. The larger variation of MUC5B viscosity values in the presence of EGCG compared to untreated MUC5B solutions, particularly at high mucin concentrations, suggests heterogeneity in the EGCG-MUC5B mixtures. The heterogeneity of MUC5B and EGCG mixtures was examined further using AFM to visualise the changes to the MUC5B network.

**Figure 4:**
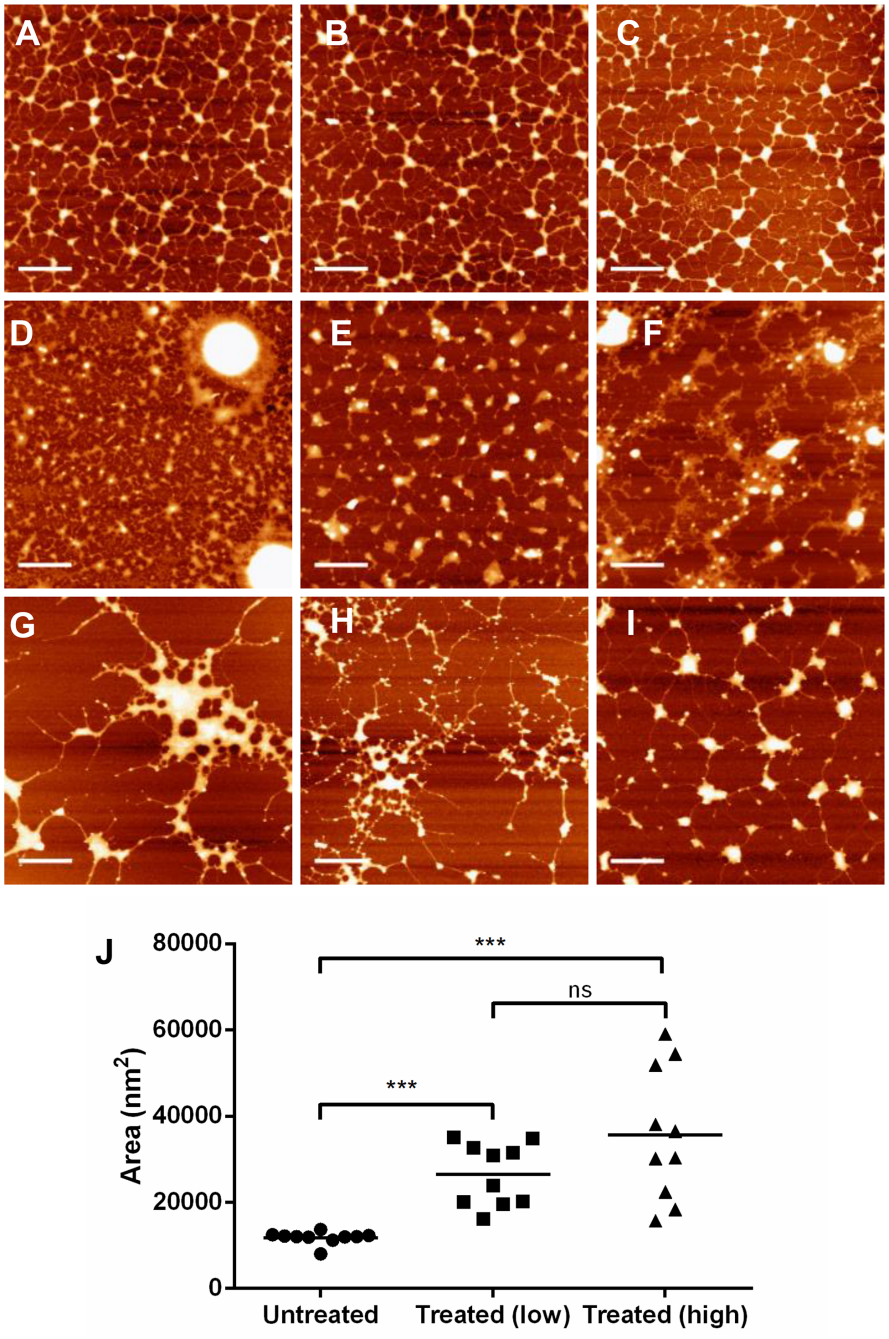
AFM reveals alteration to the MUC5B network by EGCG. MUC5B at a concentration of 15 µg/mL was imaged with PBS (A–C), 75 µM EGCG (D–F) and 375 µM EGCG (G–I) on APTES-coated mica. Representative images are shown. Scale bars represent 1 µm in all cases. The height scale of the images is 4 nm (A–C), 9 nm (D), 7 nm (E), 10 nm (F), 6 nm (G, I) and 5 nm (H). J: Mean area of aggregates above 1 nm in height. AFM images of MUC5B with PBS (untreated, circles), 75 µM EGCG (treated low-dose, squares) and 375 µM EGCG (treated high-dose, triangles) were quantified using Nanoscope software (n = 10). Particles above a height threshold of 1 nm were identified and their area quantified, the mean is plotted here. The variation seen in the presence of EGCG suggests heterogeneity of aggregation with increasing EGCG concentration. Parametric paired t-tests were performed to determine signficance. Ns = not significant. *** p value 0.0001–0.001.

In the absence of polyphenol, MUC5B forms a network, as shown in Figure 4 A–C. At 15 µg/mL, the untreated MUC5B network shows some aggregation on the APTES-coated surface, highlighted by the large blobs at a higher height scale than the fibrillar mucin strands they are connected with. This network structure of untreated MUC5B was representative and seen in all images captured.

The change of the MUC5B network in the presence of EGCG (Figure 4 D–F) was not uniform. In some areas there was a more densely packed network and the presence of large aggregates (D), in others there was a similar pore-size as untreated MUC5B but with larger aggregates (E), and in other regions there were larger pore-sizes with large aggregates (F). In all cases there was an increase in molecular height, suggesting aggregation. The aggregation was also altered when higher concentrations of EGCG were added (Figure 4 G–I), with generation of very large aggregates with few connecting mucin strands (G), smaller aggregates with many connecting strands (H) and in some places networking was observed but with high aggregation (I). Images were quantified to examine the level of aggregation.

For quantification of aggregation, a height threshold of 1 nm was chosen; all regions above a height of 1 nm were classed as aggregates. This is due to the observation that the mucin threads that link the larger blobs in untreated MUC5B images were approximately equal to or less than 1 nm in height. Using Nanoscope Analysis software, aggregates above a height threshold of 1 nm were identified, their area quantified, and the mean calculated. The mean area of aggregates in untreated MUC5B was very similar in all images analysed (Figure 4 J). Upon addition of EGCG the mean area of aggregates increased in a dose-dependent manner. Furthermore, the variation in aggregate structure increased substantially upon addition of increasing concentrations of EGCG, highlighting the heterogeneity of changes to MUC5B.

While these data clearly show an altered MUC5B network caused by EGCG, it is noteworthy that MUC5B was not fully precipitated by EGCG, and that molecules could still be seen to be connected to one another via threads/strands in Figure 4 D–I. This suggests that some regions of MUC5B may not be involved in the aggregation process, and prevent the entire molecule from being precipitated. To investigate this further, we isolated the highly glycosylated regions of MUC5B (T-domains, generated by trypsin digestion), and expressed recombinant N-terminal and C-terminal protein domains of MUC5B, and examined their interaction with EGCG. In these experiments, a higher concentration of 4 mM EGCG was used to maximise the extent of the interaction to better demonstrate whether EGCG causes a change in the aggregation state of these components.

### The protein domains of MUC5B are aggregated by EGCG

**Figure 5:**
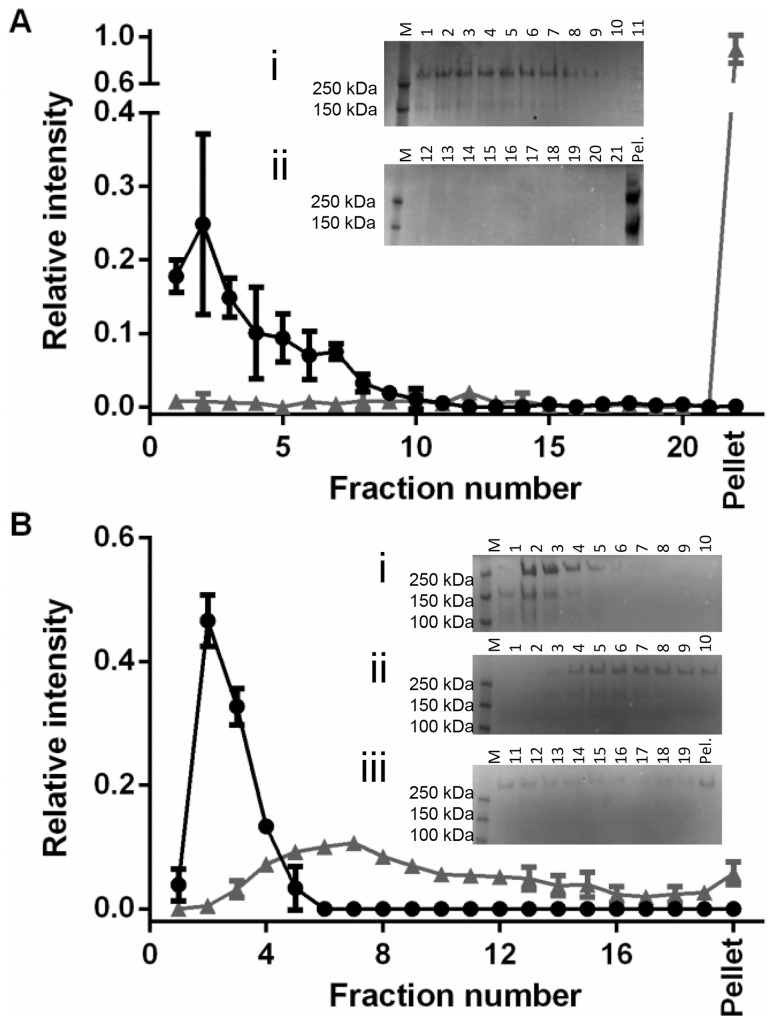
Aggregation of MUC5B protein domains by EGCG. (A) Rate-zonal centrifugation of 380 µg/mL NT5B recombinant protein (D1-D2-D′-D3 domains of MUC5B) in PBS (black circles) or 4 mM EGCG (grey triangles) in 5–20% (w/v) sucrose gradients (n = 2). Sucrose gradients were fractionated into 21 fractions and pelleted material was solubilised in urea. Fractions were run on SDS-PAGE gels and silver stained. NT5B is expressed as a monomer and a dimer. Inset (i) shows the SDS-PAGE gel of the first 11 fractions of NT5B in PBS sucrose gradient, showing all NT5B was contained within the first 11 fractions. Inset (ii) shows the SDS-PAGE gel of fractions 12–21 and the pellet of an NT5B with EGCG gradient, showing that in the presence of EGCG all NT5B is pelleted to the bottom of the tube. (B) Rate-zonal centrifugation of 500 µg/mL CT5B recombinant protein (D4-B-C-CK domains of MUC5B) in PBS (black circles) or 4 mM EGCG (grey triangles) in 5–20% (w/v) sucrose gradients (n = 2). Gradients were fractionated into 19 fractions and pelleted material was solubilised in urea. Fractions were run on SDS-PAGE gels and Coomassie stained. CT5B is expressed as a monomer and a dimer. Inset (i) shows the SDS-PAGE of the first 10 fractions of a sucrose gradient of CT5B in PBS, showing all CT5B was present in the first 5 fractions. Insets (ii) and (iii) show the SDS-PAGE gels of fractions 1–10, and 11–19 and the pellet, respectively, showing that in the presence of EGCG, there is faster sedimentation of CT5B but not full aggregation. Stained SDS-PAGE gels were imaged on a Biorad ChemiDoc MP Imaging System to measure band intensities. Pel. = pelleted material.

An N-terminal construct of MUC5B containing D1-D2-D′-D3 domains (NT5B) was stably transfected into 293-EBNA cells and the recombinant protein isolated and purified as both a monomer and a dimer (75 and 150 kDa, respectively) [40]. Rate-zonal centrifugation on 5–20% (w/v) sucrose gradients showed untreated NT5B was present at the top of the gradient (fractions 1–11) (Figure 5 A). Using band intensities from SDS-PAGE in Figure 5 A, the percentage of NT5B in fractions across the sucrose gradient was calculated (Table 1). This highlighted that 76±2.29% of untreated NT5B was present at the top of the gradient in fractions 1–5, and 20.72±4.31% was present in fractions 6–10. There was very little NT5B detected in later fractions of the gradient.

**Table 1:** Distribution of untreated and EGCG-treated NT5B across the sucrose gradient.

Rate-zonal fractions	Percentage of NT5B in rate-zonal fractions	
	Untreated (n = 2)	EGCG-treated (n = 2)
1–5	76.83±2.29	2.61±3.69
6–10	20.72±4.32	3.47±4.9
11–15	0.89±0.18	3.91±2.24
16–21	1.46±2.07	0.82±1.17
Pellet	0.09±0.13	89.19±12

SDS-PAGE band intensities were used to determine the percentage of NT5B in fractions across the sucrose gradients of NT5B +/−4 mM EGCG. Gradients were performed in duplicate and the mean values are shown, as well as standard deviation.

Upon addition of EGCG, rate-zonal centrifugation resulted in full aggregation of the entire NT5B population, with the major proportion of NT5B present in the pellet at the bottom of the tube, as shown in Figure 5 A. This is also highlighted in Table 1, which shows the percentage of NT5B in fractions across the EGCG-treated sucrose gradient. This revealed that 89.19±12% of the NT5B was pelleted in the presence of EGCG under these conditions.

A C-terminal construct of MUC5B containing D4-B-C-CK domains (CT5B) was expressed and purified using the same methods as for NT5B, and yielded a mixture of monomer and dimer (160 and 280 kDa, respectively). Rate-zonal centrifugation under the same conditions used for NT5B showed that untreated CT5B was present at the top of the gradient, in fractions 1–5 (Figure 5 B). Using band intensities from SDS-PAGE in Figure 5 B, the percentage of CT5B in fractions across the sucrose gradient was determined (Table 2). This demonstrated that 100% of untreated CT5B was present at the top of the gradient in fractions 1–5.

**Table 2:** Distribution of untreated and EGCG-treated CT5B across the sucrose gradient.

Rate-zonal fractions	Percentage of CT5B in rate-zonal fractions	
	Untreated (n = 2)	EGCG-treated (n = 2)
1–5	100±0	20.04±4.08
6–10	0±0	41.73±0.03
11–15	0±0	23.14±5.99
16–19	0±0	9.31±0.02
Pellet	0±0	5.77±1.86

SDS-PAGE band intensities were used to determine the percentage of CT5B in fractions across the sucrose gradients of NT5B +/−4 mM EGCG. Gradients were performed in duplicate and the mean values are shown, as well as standard deviation.

Upon addition of EGCG, there was a substantial change to the sedimentation profile of CT5B (Figure 5 B). CT5B was not fully aggregated, but was broadly distributed throughout the sucrose gradient. As shown in Table 2, using band intensities from SDS-PAGE in Figure 5 B, the percentage of CT5B in fractions across the sucrose gradient was determined. This showed that in the presence of EGCG, only 20±4.08% of CT5B was present at the top of the gradient, in fractions 1–5. This is in sharp contrast to untreated CT5B, in which the entire population of CT5B was at the top of the gradient. The majority (41.73±0.03%) of CT5B treated with EGCG was present in fractions 6–10, while 23.15±5.99% was present in fractions 11–15, 9.31±0.02% in fractions 16–19 and 5.77±1.86% in the pellet. These data suggest the heterogeneous reorganisation of CT5B by EGCG and the formation of a broad distribution of protein aggregates under these conditions. The same rate-zonal centrifugation conditions were used to test the effect of EGCG on trypsin resistant, oligosaccharide-rich regions (T-domains), which are derived from the central mucin domain of MUC5B.

### MUC5B oligosaccharide-rich regions are not aggregated by EGCG

**Figure 6:**
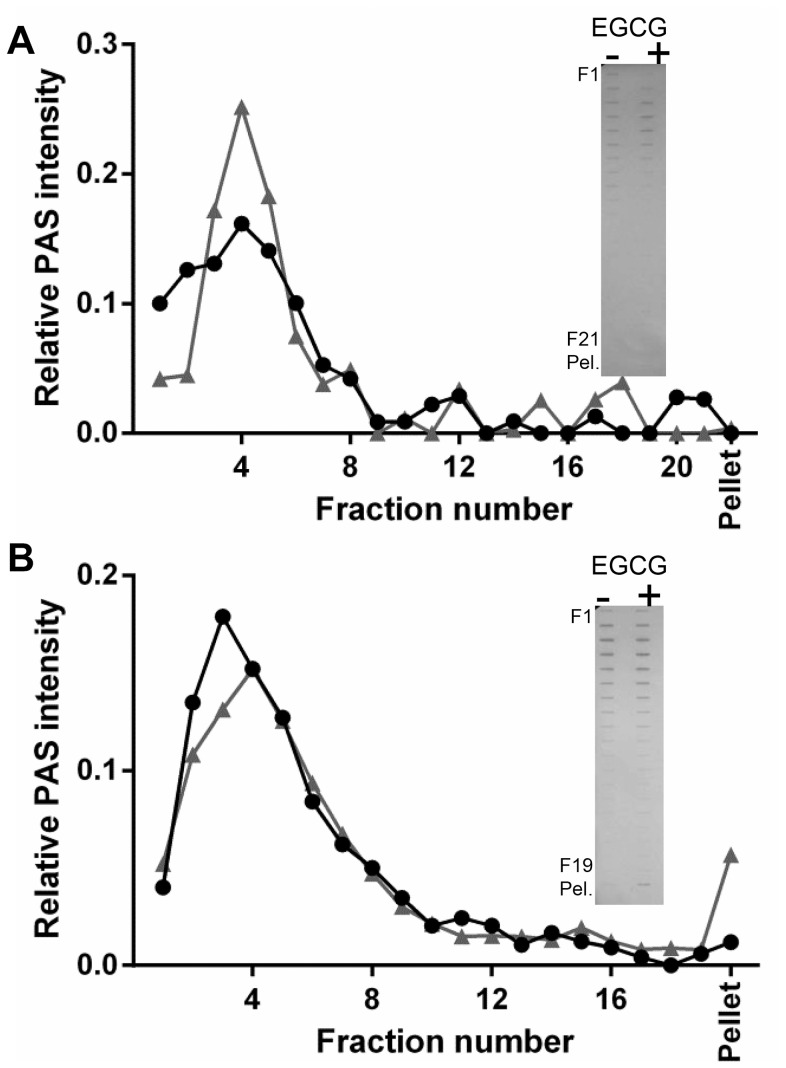
MUC5B oligosaccharide-rich regions do not aggregate in the presence of excess EGCG. MUC5B T-domains were generated by trypsin-digestion and made to 200 µg/mL (A) and 600 µg/mL (B) before rate-zonal centrifugation with PBS (black circles) or 4 mM EGCG (grey triangles), in 5–20% (w/v) sucrose gradients. EGCG was removed from fractions using a HiTrap desalting column, fractions were slot blotted and PAS stained to detect glycoprotein (inset). Blots were scanned using Biorad ChemiDoc MP Imaging System and intensities measured using ImageLab software.

MUC5B T-domains were isolated by trypsin digestion of reduced and alkylated purified MUC5B. The T-domains of MUC5B were tested for an interaction with EGCG under the same conditions as for NT5B and CT5B (on 5–20% (w/v) sucrose gradients). With EGCG in excess, rate-zonal centrifugation showed almost no change in the sedimentation profile of 200 µg/mL T-domains and only a minor proportion became insoluble at 600 µg/mL T-domains (Figure 6 A and B respectively). While it cannot be ruled out that there is an interaction between MUC5B T-domains and EGCG, the data presented here demonstrate that such an interaction does not lead to aggregation of T-domains. In addition to our investigations of salivary MUC5B, we studied the role of the smaller non-gel-forming salivary mucin, MUC7, in the interaction with EGCG.

### Non-gel-forming mucin MUC7 is precipitated by EGCG

**Figure 7:**
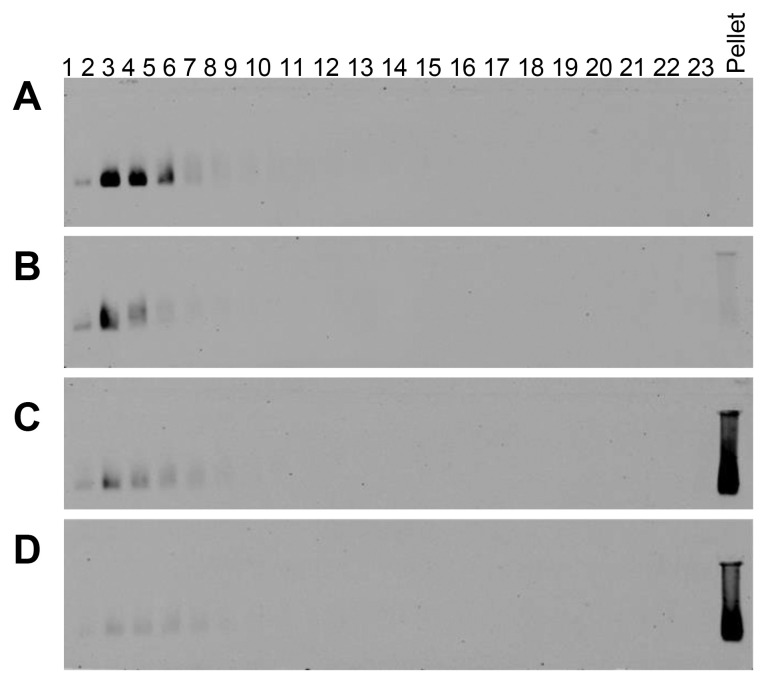
Aggregation of MUC7 in human whole saliva by EGCG. Agarose gel electrophoresis of rate-zonal centrifugation fractions of human whole saliva with (A) PBS, (B) 100 µM EGCG, (C) 500 µM EGCG and (D) 1 mM EGCG in 10–50% (w/v) sucrose gradients, probed with EUMUC7a and developed using the Odyssey Imaging system.

**Figure 8:**
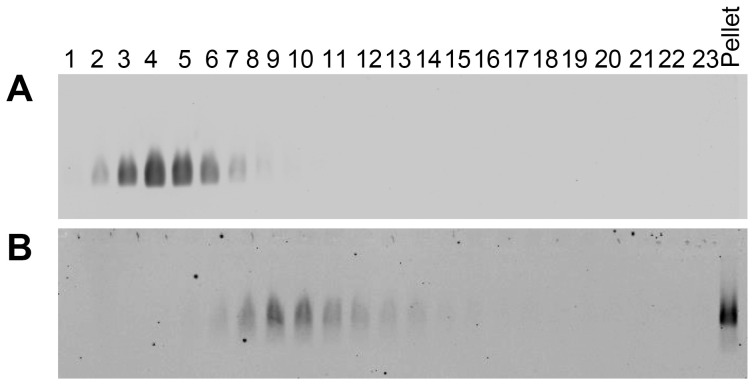
MUC7, purified by isopycnic density gradient centrifugation, interacts with EGCG. Agarose gel electrophoresis of rate-zonal centrifugation fractions of 115 µg/mL purified MUC7 in 5–20% (w/v) sucrose gradients with PBS (A) or 4 mM EGCG (B). Gels were transferred to nitrocellulose, probed with the EUMUC7a anti-MUC7 antibody and developed using Odyssey Imaging software.

Human whole saliva was subjected to rate-zonal centrifugation in 10–50% (w/v) sucrose gradients, before and after incubation with EGCG and probed for MUC7 (Figure 7). In untreated saliva, MUC7 was present at the top of the gradient (fractions 2–5), as shown in Figure 7 A. At concentrations of EGCG as low as 100 µM, a small proportion of MUC7 was pelleted (Figure 7 B). The amount of MUC7 pelleted increased with increasing EGCG concentration, with almost all MUC7 pelleted in the presence of 1 mM EGCG (the approximate concentration of EGCG in green tea), (Figure 7 D). After purification of MUC7 by isopycnic density gradient centrifugation, EGCG substantially changed the sedimentation profile of MUC7, causing at least half of the mucin population to become insoluble (Figure 8).

### Polymeric MUC5B and non-gel-forming MUC7 are not aggregated by EC

**Figure 9:**
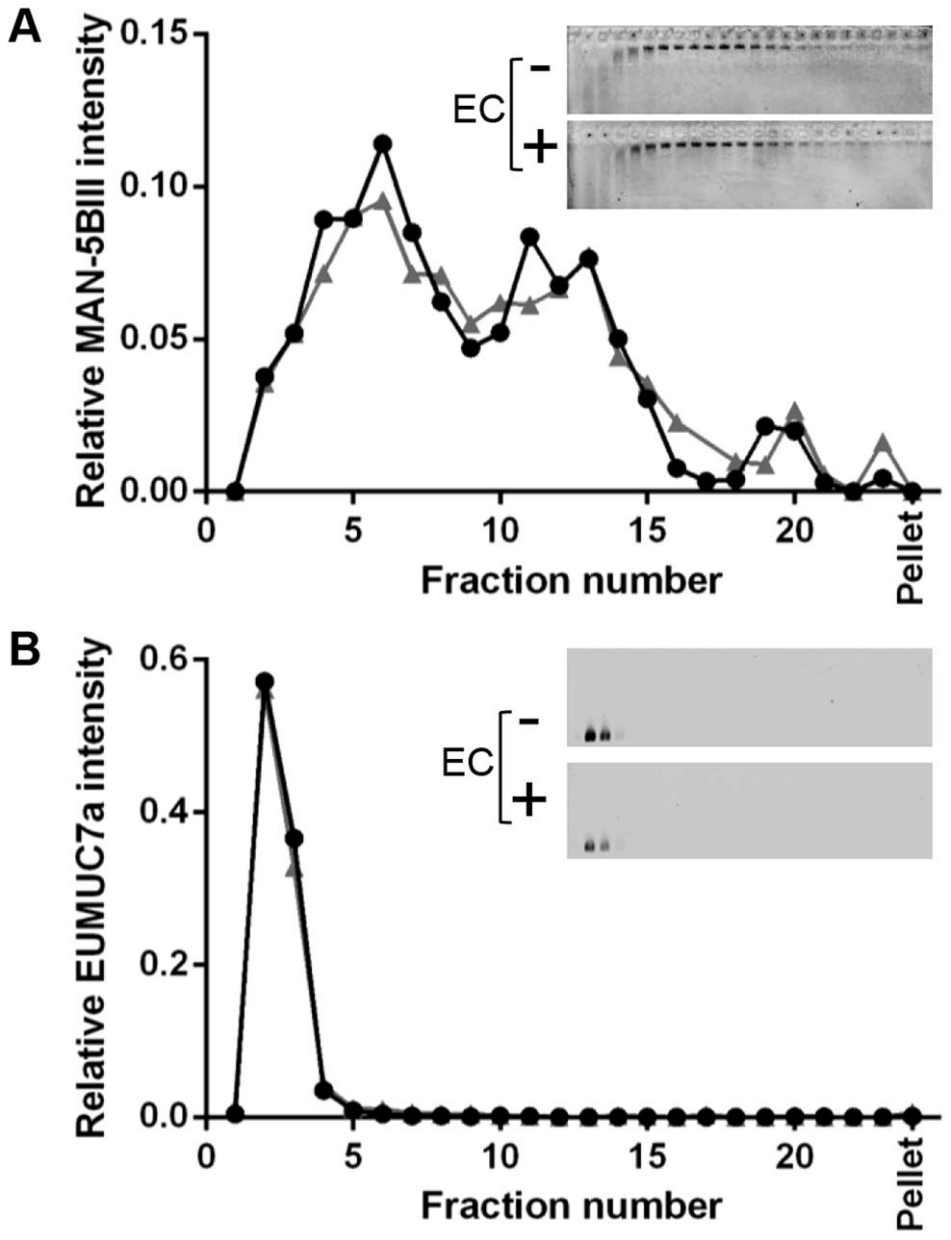
EC does not cause aggregation of salivary mucins. Rate-zonal centrifugation of human whole saliva with 1 mM EC (grey triangles) or PBS (black circles), performed in 10–50% (w/v) sucrose gradients in PBS pH 7.4. (A) MUC5B was detected by agarose gel electrophoresis of rate-zonal fractions and western blotting with the MAN-5BIII antiserum (inset; saliva (top panel) and saliva+EC (bottom panel)). (B) MUC7 was detected by agarose gel electrophoresis of rate-zonal fractions and western blotting with the EUMUC7a antiserum (inset; saliva (top panel) and saliva+EC (bottom panel)). Bands were quantified using the Odyssey Imaging system.

While EGCG is the most abundant polyphenol in green tea, other polyphenols such as EC also contribute to the astringency of green tea. Therefore, the effect of EC on salivary mucins was explored here. The concentration of EC in green tea is reported to be 100–272 µM [57], but a final concentration of 1 mM was used here to compare with the concentration used for EGCG. Human whole saliva was mixed with a final concentration of 1 mM EC, subjected to rate-zonal centrifugation (on 10–50% (w/v) sucrose gradients) and analysed by agarose gel electrophoresis and western blotting with MUC5B and MUC7 antisera (Figure 9).

In the absence of EC, MUC5B was present in fractions 4–15 (Figure 9 A). Upon addition of EC, there was no detectable change in the sedimentation profile of MUC5B. When fractions were detected with MUC7 antiserum (Figure 9 B), in the absence of EC MUC7 was present at the top of the gradient (fractions 2–4). In the presence of EC, there was no change in the sedimentation behaviour of MUC7. These data suggest that, unlike EGCG, EC does not cause aggregation of MUC5B or MUC7.

## Discussion

This study has shown how the main polyphenol in green tea, EGCG, alters the properties of both the gel-forming and non-gel-forming mucins in saliva. The majority of non-gel-forming mucin MUC7 in human whole saliva was aggregated by EGCG, and purified MUC7 also showed a substantial increase in sedimentation rate in the presence of EGCG. Furthermore, using both purified MUC5B and MUC5B in whole saliva, EGCG resulted in an increased sedimentation rate of mucin, increased mucin aggregation and increased mucin viscosity. It was revealed that EGCG caused these effects by binding predominantly to the protein domains of MUC5B, causing their aggregation. In contrast, another green tea polyphenol, EC, did not cause aggregation of either MUC5B or MUC7 in human whole saliva.

This is the first time that purified polymeric salivary mucins have been extensively studied for their interaction with green tea polyphenols and has highlighted the substantial effects that dietary compounds can have on the mucin network. Elsewhere, there have been conflicting reports regarding the aggregation of salivary mucins by polyphenols, but such studies have been restricted by the use of poorly characterised commercial mucins or the use of techniques such as SDS-PAGE in which the migration of mucins is limited [28], [32]–[37], [58], [59]). Therefore, the contribution of salivary mucins to the interaction with EGCG was somewhat unclear. By utilising well characterised polymeric mucins, purified under native conditions, alongside techniques such as rate-zonal centrifugation coupled with agarose gel electrophoresis to resolve high molecular weight biopolymers, we have shed light on the substantial alteration to the salivary mucin networks by the green tea polyphenol EGCG.

It is widely regarded that the aggregation of other salivary proteins (proline-rich proteins, cystatins, histatins and amylase) by EGCG contributes to the astringency of green tea [23]–[29]. Therefore, the interactions reported here for MUC5B and MUC7 with EGCG may also contribute to the dry sensation in the oral cavity upon consumption of green tea. The alteration of the MUC5B network by EGCG may also reduce the lubrication properties of the gel-forming mucin, offering another possible mechanism that may contribute to green tea astringency.

We have shown that the effects of EGCG on mucins are exacerbated at higher MUC5B concentrations. This may suggest that in regions of the oral cavity where mucin concentration is highest, such as the oral mucosal pellicle and salivary film that line the soft tissues of the mouth, the consequences of EGCG may be greatest [55], [56], [60], [61]. If interactions between mucins and EGCG do contribute to astringency, this could suggest that astringency may be caused by changes to the oral mucosal pellicle, rather than by changes to whole saliva. Indeed, it has been reported elsewhere that removal of saliva by rinsing of the oral cavity caused an increase in the astringency perception of tea, suggesting that the oral mucosal pellicle is important for the detection of astringency [62]. The sites of mucin aggregation by EGCG were also examined here.

EGCG caused aggregation of the N-terminal region of MUC5B (D1-D2-D′-D3 domains) and the formation of a broad distribution of aggregates of the C-terminal region of MUC5B (D4-B-C-CK domains). In sharp contrast, the oligosaccharide-rich regions of MUC5B were not aggregated. In similar observations, it has been reported elsewhere that glycosylation of proline-rich proteins reduces their aggregation by EGCG [64], [65]. While the direct binding site for EGCG on mucins was not investigated here, it is thought that hydrogen bonding and hydrophobic interactions may be involved [37], [66]. Furthermore, studies on PRPs have suggested that proline residues are particularly important for EGCG binding. Proline residues are enriched in the heavily-glycosylated regions of mucins but their interaction with EGCG is likely stopped by the hydrophilic O-glycans. Other residues, in addition to proline, including arginine, phenylalanine and histidine, are also important in binding to polyphenols [67]. All of these residues are found within the N-terminal and C-terminal domains of MUC5B and MUC7 (accession numbers Q9HC84.2 and Q8TAX7, respectively) and so there are likely to be many binding sites for EGCG. This has been observed for PRPs in which 8 EGCG molecules may bind to a single PRP molecule [68]. The region of EGCG that is involved in this binding has not been determined, but since EC (which lacks a galloyl ring) did not cause precipitation of MUC5B and MUC7 in human whole saliva, the galloyl ring of EGCG may be important. Similarly, epigallocatechin (EGC) also lacks a galloyl ring and so may be predicted to not cause mucin aggregation. The lack of changes to MUC5B and MUC7 by EC is in agreement with observations elsewhere that EC does not alter the rheological properties of saliva, while EGCG does [20], [22]. This suggests that EC causes astringency by alternative mechanisms [20].

The ability of EGCG to cause aggregation is due to its multidentate properties and ability to self-associate, thus bridging two proteins together [27], [69]. Therefore, a model can be proposed whereby EGCG, via its galloyl ring, causes aggregation of MUC5B by bridging the exposed protein backbone that is not protected by dense glycan chains, leaving glycan chains exposed. In the case of mucins, since glycans have such an important role in the gel formation of mucins [70], their ability to avoid aggregation ensures maintenance of the gel integrity. In the mucin network, it may be possible that this aggregation of protein domains by EGCG may create greater exposure of mucin glycan chains to the local microenvironment, facilitating the binding and clearance of pathogens by mucins, and increasing the repertoire of pathogens to which salivary MUC5B may bind. While MUC5B is the gel-forming mucin in saliva, the smaller non-gel forming mucin MUC7 is also secreted in saliva and was studied in this report.

Both native and purified MUC7 from saliva interacted with EGCG. Furthermore, in human whole saliva, MUC7 organisation seemed to be much more affected than MUC5B by EGCG. This suggests that complexes of MUC7 with other proteins in saliva may contribute to its aggregating ability [9], [10]. Indeed, MUC7 is aggregated under many different circumstances, as has been highlighted by the self-association of MUC7 under non-denaturing conditions [31] and its formation into micelles with other salivary proteins [10], as well as the aggregation of bacteria by MUC7 [5], [6]. Additionally, differences in the structure of the mucins may contribute to the different effects by EGCG, since it is reported that MUC7 has shorter glycan chains and less dense glycosylation compared to MUC5B [71], [72]. It has also been reported recently that the effect of EGCG on the mucins is not limited to the mucins expressed in the oral cavity [38].

It has been shown that polymeric gastrointestinal mucins have increased viscosity in the presence of EGCG [38]. Although these interactions may sequester EGCG, reducing its intestinal absorption and limiting its bioavailability, it is possible that the interactions of EGCG with the mucin networks of the digestive tract may provide a mechanism to retain the polyphenol and maximise its health benefits. Indeed, studies on human salivary acinar cells have shown that EGCG may protect salivary glands from cytotoxicity, and in pathologies such as Sjogren’s syndrome EGCG may provide protection against autoimmune-induced tissue damage [73]. Furthermore, various studies have shown that green tea polyphenols may inhibit tumorigenesis throughout the gastrointestinal tract, as reviewed by Yang *et al*. [74]. It is also possible that the interactions of mucins with EGCG may facilitate the weight loss benefits of green tea, since the altered mucosal barriers may restrict the passage and absorption of other nutrients, reducing calorie intake. As discussed here, green tea polyphenols, particularly EGCG, are important dietary components, however, other dietary compounds are also likely to have an influence on mucosal barriers throughout the gastrointestinal tract.

Other galloylated polyphenols in plant-derived foods and beverages may affect saliva and mucosal barriers in a similar way to EGCG. Indeed grape seed extract has been shown to interact with commercially available bovine submaxilliary mucin [35], [36], demonstrating a mechanism by which red wine consumption may have comparable effects on mouth-feel. In addition to polyphenols, acids have also been observed to precipitate mucins [33]. Another example of mucin-binding dietary compounds are plant lectins, present in seeds, nuts, potatoes and beans, which have the ability to bind to the oligosaccharides of mucins and in this way, cross-link mucins into multilayers [75]. This is in sharp contrast to the polyphenol binding to mucin protein domains in this study, but demonstrates how different molecules act on diverse salivary substrates. Dietary components change the barrier properties of mucous secretions in different ways. Here, we highlight how one dietary molecule, EGCG, can alter the salivary mucins by aggregating their protein domains and altering their network properties.
